# TransGrid-CostOpt: A hybrid transformer framework for cost prediction and optimization of distribution network assets

**DOI:** 10.1371/journal.pone.0350026

**Published:** 2026-05-29

**Authors:** Wei Xiong, Jie Xia, YiBo Yu, SanMing Xiong, HaiYang Hu, Peng Wan, Dan Li

**Affiliations:** 1 State Grid Hubei Electric Power Co., Ltd, Wuhan, Hubei, China; 2 State Grid Hubei Electric Power Co., Ltd. Jingzhou Power Supply Company, Jingzhou, Hubei, China; 3 Wuhan Anju Supply Chain Co., Ltd, Wuhan, Hubei, China; 4 International Education College of North China Electric Power University (Baoding), Baoding, Hebei, China; 5 Hubei Power Grid Design Co., Ltd., Wuhan, Hubei, China; 6 Yunxia Electric Energy (Yunnan) Co., Ltd, Kunming, Yunnan, China; University of Lagos Faculty of Engineering, NIGERIA

## Abstract

The prediction and optimization of distribution network asset costs is a complex problem in the power industry, involving the optimization of multiple objectives and the response to dynamic demands. Traditional methods often struggle to effectively address fluctuations in power load and the uncertainties in the supply chain, limiting their effectiveness in complex environments. To solve this issue, we propose the TransGrid-CostOpt model, an intelligent cost optimization model that integrates deep learning, multi-objective optimization, time-series forecasting, and optimization decision-making modules. TransGrid-CostOpt optimizes load forecasting and cost allocation for the distribution network by combining multi-source data, time-series load forecasting, and reinforcement learning decision strategies, reducing operational costs, improving load forecasting accuracy, and enhancing decision adaptability. Experimental results show that TransGrid-CostOpt outperforms traditional models and other advanced methods on the BuildingsBench and PJM Hourly Load Data datasets, exhibiting higher accuracy and efficiency in forecasting, cost optimization, and multi-objective balancing. Compared to classical baseline models and cutting-edge approaches, TransGrid-CostOpt demonstrates a 15% to 30% overall performance improvement. Ablation experiments confirm the critical role of each module, especially the time-series forecasting module and optimization decision-making module, in significantly enhancing the model’s performance. TransGrid-CostOpt strengthens the cost management capability of the distribution network and shows strong adaptability in dynamic electricity market environments, with broad application potential.

## Introduction

With the rapid development of the power industry, the distribution network is playing an increasingly important role in the power system. The distribution network is not only a channel for power transmission but also involves multiple tasks such as asset management, equipment maintenance, and load forecasting [1]. In the management of electrical equipment and distribution facilities, accurately predicting and optimizing the cost of distribution network assets has become a key issue that needs to be addressed. With the liberalization of the electricity market, cost control has become a crucial factor for power companies to maintain a competitive edge [2,3]. Therefore, the cost optimization of distribution network assets not only helps to improve the operational efficiency of power companies but also enhances the overall reliability and economic performance of the power system [4,5].

In past research, numerous methods have been proposed to address the problem of cost prediction and optimization in the distribution network [6]. Some studies are based on traditional regression analysis methods, such as linear regression, multiple regression, and ridge regression, to establish the relationship between costs and various factors [7]. Another category of research uses statistical methods, such as time series analysis and regression trees, to handle dynamic data and forecast future trends [8]. Additionally, simulation and optimization algorithms, such as genetic algorithms, particle swarm optimization, and simulated annealing, have been widely applied to seek optimal solutions for cost minimization [9]. With the advancement of machine learning, some ensemble learning methods, such as random forests and gradient boosting trees, have also been introduced into the optimization of distribution networks. These methods can effectively handle nonlinear relationships and improve the accuracy of predictions [10]. Deep learning methods, such as CNNs, have also begun to be applied to cost prediction, particularly demonstrating strong capabilities when handling large-scale high-dimensional data [11]. Despite these advancements, existing methods still face considerable challenges in dynamic load fluctuations and high-uncertainty environments. The optimization of distribution networks has seen significant advancements through specialized applications in critical sectors. Recent contributions highlight this trend, with a scientometric review providing a foundational understanding of the blood supply chain, identifying key trends and the intellectual structure of this life-saving logistics network [12]. Extending such optimization principles to high-stakes environments, a bi-objective model for managing resources in a smart hospital has been introduced, explicitly balancing operational costs with user preferences. Together, these works underscore the field’s evolution towards more complex, multi-objective, and context-sensitive distribution models [13].

This paper proposes a novel hybrid Transformer-based framework, TransGrid-CostOpt, for cost prediction and optimization of distribution network assets. By introducing Transformer into traditional cost prediction and optimization tasks, our method addresses several key issues in traditional approaches by incorporating various data features. Specifically, this paper designs a deep learning framework that integrates time-series data, component features, and external economic factors to improve the accuracy of cost prediction and the capability of decision optimization for distribution networks. The core of the framework consists of three modules: Feature Extraction and Fusion Module, which utilizes a Transformer encoder to extract global deep features and employs MLP for multi-source data fusion and dimensionality reduction; Time Series Forecasting Module, which uses bidirectional LSTM to capture long- and short-term dependencies in time-series data for multi-step load and cost forecasting; and Optimization Decision Module, which leverages Hierarchical Meta-Reinforcement Learning for dynamic decision optimization, enhancing the model’s adaptability in responding to changing environments. The three main contributions of this paper are:

1. A Transformer-based framework for distribution network asset cost prediction and optimization is proposed.
2. By integrating multi-source heterogeneous data, the model’s accuracy and adaptability are enhanced.
3. Cost decisions are optimized through reinforcement learning, demonstrating the feasibility and advantages of the model in practical applications.

## Related work

### Cost prediction and optimization of distribution network

In the field of distribution network cost prediction and optimization, numerous methods have emerged in recent years. As the complexity of power systems increases, finding a balance between cost control, resource allocation, and system stability has become a core research issue [14]. Traditional optimization methods have achieved certain results in solving simple problems, but they still have limitations when faced with factors such as fluctuating load demands, equipment aging, and policy adjustments [15]. To address these challenges, an increasing number of innovative methods have been proposed and gradually applied to the optimization of real-world distribution networks. Some studies use Bayesian networks and Gaussian process regression (GPR) to model relationships between complex data and improve prediction accuracy through probabilistic inference [16]. Genetic algorithms (GA) and ant colony optimization (ACO) have been widely applied to optimize the equipment configuration and cost minimization of distribution networks, showing good performance in multi-objective optimization [17]. Adaptive weighted averaging algorithms (AWM) and fuzzy logic systems (FLS) handle uncertainty and fuzzy data by adjusting input feature weights, with good applications in distribution network forecasting. Methods based on GNNs have also started to gain attention, leveraging graph-structured data to model the complex relationships between devices and networks [18]. Although these methods have made progress in certain scenarios, challenges such as insufficient adaptability and poor real-time performance remain when dealing with multi-dimensional data and complex dynamic environments [19,20].

Unlike these methods, the TransGrid-CostOpt model proposed in this paper employs a Transformer-based hybrid framework, capable of handling complex data from different sources, integrating time-series and external economic factors for prediction, and incorporating reinforcement learning to optimize cost decisions. These improvements enable our model to provide more accurate and efficient solutions when facing the complex and dynamic environment of distribution networks.

### Distribution network optimization: Applications and limitations

In the field of distribution network optimization, numerous methods have been proposed and applied to various optimization tasks. As the complexity and scale of distribution networks increase, optimization problems are becoming increasingly challenging, especially in the context of multi-objective, multi-constraint, and time-varying data [21,22]. To enhance system efficiency and economic performance, scholars have proposed various optimization methods covering aspects such as load scheduling, equipment configuration, cost minimization, and system stability improvement. Optimal Power Flow (OPF) has been widely used in load scheduling and power distribution of distribution networks, aiming to enhance overall system efficiency by minimizing transmission losses and optimizing operational costs [23]. Multi-objective optimization (MOO) methods consider multiple optimization objectives simultaneously, such as cost reduction, system stability, and minimizing environmental impact, and are commonly applied to resource scheduling and planning issues in distribution networks [24]. Dynamic Programming (DP) and Rolling Optimization (RO) are used for phase-based scheduling and decision optimization based on real-time data [25]. In data-driven optimization methods, Clustering Analysis and Principal Component Analysis (PCA) are used for dimensionality reduction and feature extraction, helping to simplify the processing of high-dimensional data. RL has shown excellent performance in load forecasting, equipment management, and system adaptive optimization, achieving significant results in some dynamic optimization tasks [26] . Although these methods have addressed some issues in distribution network optimization, they still face challenges in handling multi-source heterogeneous data, coping with real-time changes, and improving computational efficiency [27].

Unlike these methods, the TransGrid-CostOpt model proposed in this paper combines the powerful time-series modeling capabilities of the Transformer and optimizes cost decisions through reinforcement learning, enabling the model to efficiently process multi-source heterogeneous data and provide precise decision support in complex, dynamically changing environments. With this innovative design, TransGrid-CostOpt demonstrates superior advantages in terms of accuracy, adaptability, and real-time performance for cost prediction and optimization.

## Method

### Ethics statement

This research did not involve human participants, animals, or any data collected from private individuals in a manner requiring ethical approval. All data used in this study are publicly available, anonymized, and intended for academic research purposes.This work was conducted in accordance with standard academic integrity practices to ensure the reproducibility and transparency of the research.

### Overview of our network

**Fig 1:**
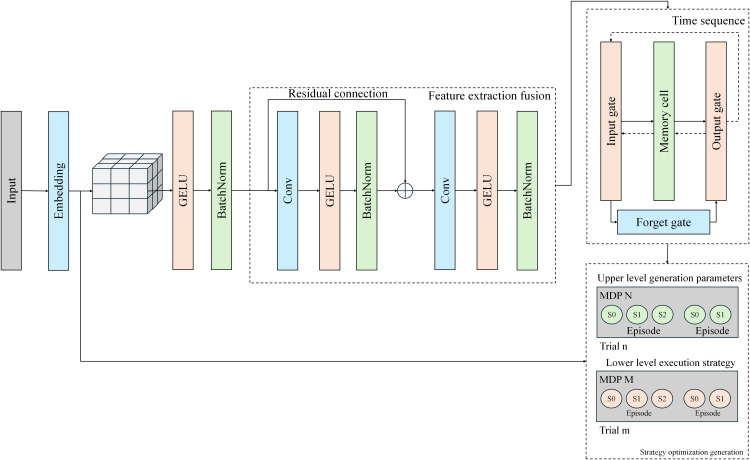
TransGrid-CostOpt: Architecture Diagram of a Hybrid Framework for Cost Prediction and Optimization of Distribution Network Assets.

The TransGrid-CostOpt model proposed in this paper addresses the problem of distribution network asset cost prediction and optimization through a multi-level data processing and decision optimization framework. The core of the model lies in the integration of different types of data, and through the collaborative work of modules such as feature extraction, time-series forecasting, and optimization decision-making, it achieves adaptation and optimization for complex distribution network environments. The overall structure of the model is shown in Fig 1, which illustrates the functions of each module and the data flow.

In the feature extraction and fusion module, the raw time-series data and static device attributes are input into the Transformer encoder to extract global deep features [28]. The Transformer plays a crucial role in capturing long-term dependencies and multidimensional features in the time-series data. Meanwhile, the MLP, as a supplementary component, is responsible for integrating, dimensionality reducing, and enhancing features from different sources and forms, generating the final fused features. For different types of data, we apply different processing methods: time-series data is embedded and encoded before being input into the Transformer encoder; static features are processed through a fully connected network (MLP) and fused with the time-series features; external economic factors, such as electricity prices and weather, are also transformed into a format compatible with the time-series data and ultimately fused with other data before being input into the Transformer. This process ensures the effective integration of various distribution network features, providing high-quality input data for subsequent modules. The core of the time-series forecasting module is the bidirectional LSTM, which captures both long-term and short-term dynamic changes in the time-series data for multi-step forecasting [29]. LSTM effectively leverages the sequential dependencies in historical data, demonstrating strong performance in load and cost prediction for the distribution network. The Transformer, working in conjunction with the LSTM, provides global contextual information as a supplement to the LSTM, making the prediction results more accurate and reliable. The output of this module is the forecasted load and cost values for a future period, providing critical input for the optimization decision-making module. The optimization decision-making module employs Hierarchical Meta-Reinforcement Learning (Meta-RL) to achieve dynamic optimization of distribution network costs. In this module, the prediction results and environmental states are defined as states in reinforcement learning [30]. The model generates policy parameters through the upper-level meta-policy and uses the lower-level policy to execute specific decision tasks, such as purchase quantities or scheduling instructions. Through this hierarchical structure, the model can quickly adjust its decision-making strategy with a small number of samples when facing new tasks or environmental changes, thereby improving the flexibility and efficiency of cost optimization.

Overall, the TransGrid-CostOpt model achieves the organic integration and efficient collaboration of each module through its carefully designed modular structure. Each module plays a crucial role in the data flow, from feature extraction and time-series forecasting to cost optimization decision-making, with every part of the model supporting the overall objective. Through this modular design, the model can adapt in real-time and make precise decisions when facing the complex and dynamic environment of the distribution network.

### Feature extraction and fusion for multi-source data integration

**Fig 2:**
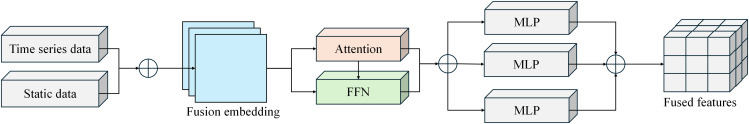
Transformer-based Multi-source Feature Fusion and Deep Representation Extraction Framework.

In the feature extraction and fusion module, the raw time-series data and static device attributes are input into the Transformer encoder to extract global deep features. The core objective of this module is to effectively capture long-term dependencies in the time-series data while integrating static attribute features, providing high-quality input data for subsequent time-series forecasting and optimization decision-making. Fig 2 illustrates the overall structure of this module, showing how the input data flows through each processing step and ultimately generates the fused features.

To achieve effective data fusion, we first applied adaptive processing to the raw time-series data and static device attributes. After the time-series data passes through an embedding layer, it is fused with static attributes via projection matrices, converting different types of data into a unified dimension. The static attributes are replicated across each time step, aligning them with the time-series data and forming a consistent input. This approach ensures that the model retains the distinctive features of each data type during processing, thus enhancing its adaptability and performance on multi-source data.

The raw multivariate time-series data $X\in {\mathbb{R}}^{L\times d_{x}}$ (where *L* is the sequence length and *d*_*x*_ is the feature dimension) is fused with the static device attributes $S\in {\mathbb{R}}^{d_{s}}$ to form an embedded representation that can be processed by the model. $W_{x}\in {\mathbb{R}}^{d_{x}\times d_{model}}$ and $W_{s}\in {\mathbb{R}}^{d_{s}\times d_{model}}$ are learnable projection matrices, and *Repeat*(*S*) denotes the operation of replicating the static attribute vector along the time steps. $b\in {\mathbb{R}}^{d_{model}}$ is the bias term. Through this operation, the time-series data and static features are transformed into a unified dimension *d*_model_, generating the initial embedded representation $H(0)\in {\mathbb{R}}^{L\times d_{model}}$:


$$\begin{array}{c} H(0)=Embedding(X,S)=XW_{x}+Repeat(S)W_{s}+b \end{array}$$ (1)

After processing through the multi-layer self-attention mechanism of the Transformer encoder, the deep features of the input data are extracted. The Transformer encoder is composed of multiple stacked layers. The input features *H*^(*n*−1)^ are transformed through linear mappings into queries (Query), keys (Key), and values (Value), and then the self-attention mechanism is used to compute the output. The output is further processed by a feed-forward neural network (FFN), followed by residual connections and layer normalization, resulting in the output *H*^(*n*)^ of the n-th layer. After processing through multiple layers of the Transformer, the final feature representation $H_{trans}=H(N)\in {\mathbb{R}}^{L\times d_{model}}$ contains global long-term dependency information, providing more enriched features for downstream tasks.


$$\begin{array}{c} {𝐐}^{\left(n\right)},{𝐊}^{\left(n\right)},{𝐕}^{\left(n\right)}={𝐇}^{\left(n-1\right)}{𝐖}_{Q}^{\left(n\right)},{𝐇}^{\left(n-1\right)}{𝐖}_{K}^{\left(n\right)},{𝐇}^{\left(n-1\right)}{𝐖}_{V}^{\left(n\right)} \end{array}$$ (2)

$$\begin{array}{c} \text{Attention}({𝐐}^{\left(n\right)},{𝐊}^{\left(n\right)},{𝐕}^{\left(n\right)})=\text{Softmax}\left(\frac{{𝐐}^{\left(n\right)}({𝐊}^{\left(n\right)}{)}^{⊤}}{\sqrt{d_{k}}}\right){𝐕}^{\left(n\right)} \end{array}$$ (3)

$$\begin{array}{c} {𝐇}^{\left(n\right)}=\text{LayerNorm}\left(\text{FFN}(\text{Attention}(\cdot ))+{𝐇}^{\left(n-1\right)}\right) \end{array}$$ (4)

The MLP (Multi-Layer Perceptron) performs fusion, dimensionality reduction, and nonlinear enhancement of the deep features output by the Transformer. *W*_*i*_ and *b*_*i*_ are the weights and biases of the i-th layer of the MLP, and σ is the activation function. While more advanced fusion techniques, such as gated fusion or cross-attention mechanisms, have been proposed in the literature, we chose the MLP fusion approach for several reasons. First, the MLP is a relatively simple and computationally efficient method that can still achieve powerful results when dealing with both static and dynamic data. It enables the model to learn nonlinear relationships between static and dynamic features without introducing unnecessary complexity. More complex methods, such as gated fusion or cross-attention, typically require additional parameters and computational resources to manage the interaction between features at different time steps. While these methods may offer improved performance in highly complex tasks, we found that the MLP approach provides an excellent trade-off between simplicity, efficiency, and model performance, especially for the forecasting and optimization tasks in our setting. In this step, the MLP module acts as a “feature palette,” selectively enhancing the feature patterns most relevant to the downstream tasks. The final output is a fused feature *H*_fused_, with unified dimensions and dense information, providing high-quality input for the time-series forecasting module.


$$\begin{array}{c} H_{fused}=MLP(H_{trans})=\sigma (\ldots \sigma (H_{trans}W_{1}+b_{1})W_{2}+b_{2}\ldots ) \end{array}$$ (5)

Through this module, the multi-source heterogeneous data of the distribution network is effectively integrated, ensuring that various data features can play a crucial role in the subsequent time-series forecasting and optimization decision-making. The design of the feature extraction and fusion module, based on capturing complex time-series dependencies and static device attributes, further enhances the model’s expressive power, laying a solid foundation for the overall performance of the model.

### Time series forecasting with bidirectional LSTM and global context enhancement

**Fig 3:**
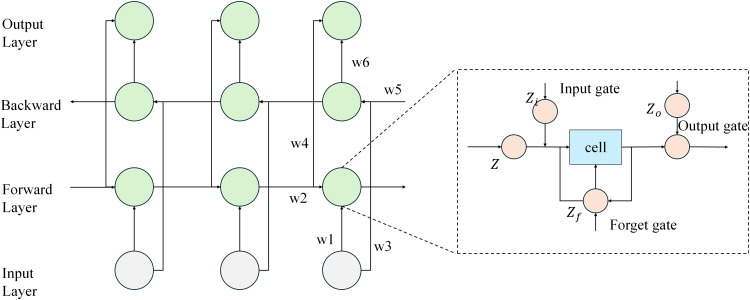
Architecture of Time Series Prediction with Bidirectional LSTM and Contextual Information Fusion.

In the time-series forecasting module, the bidirectional LSTM is the core component, capturing both long-term and short-term dynamic changes in the time-series data for multi-step forecasting. The role of this module is to predict future load and cost variations based on the sequential dependencies in historical data, providing critical input for subsequent optimization decision-making. Fig 3 illustrates the structure of this module, describing how data flows through the collaborative operation of the bidirectional LSTM and Transformer, ultimately generating forecasts for load and cost.

The bidirectional LSTM works by simultaneously learning the temporal dependencies from both past and future time-series data. The computation process of the bidirectional LSTM is divided into the forward LSTM and backward LSTM. The final hidden state *h*_*t*_ is the concatenation of the hidden states from the forward and backward LSTM.


$$\begin{array}{c} {\vec{𝐡}}_{t},{\vec{𝐜}}_{t}={LSTM}_{forward}\left({𝐇}_{fused}[t],\;({\vec{𝐡}}_{t-1},{\vec{𝐜}}_{t-1})\right),({\vec{𝐡}}_{0},{\vec{𝐜}}_{0})=0 \end{array}$$ (6)

$$\begin{array}{c} \overset{\leftarrow }{{𝐡}_{t}},\overset{\leftarrow }{{𝐜}_{t}}={LSTM}_{backward}\left({𝐇}_{fused}[t],\;(\overset{\leftarrow }{{𝐡}_{t+1}},\overset{\leftarrow }{{𝐜}_{t+1}})\right),(\overset{\leftarrow }{{𝐡}_{L+1}},\overset{\leftarrow }{{𝐜}_{L+1}})=0 \end{array}$$ (7)

$$\begin{array}{c} {𝐡}_{t}=[{\vec{𝐡}}_{t};{\overset{\leftarrow }{𝐡}}_{t}] \end{array}$$ (8)

To introduce global contextual information, the global context *c*_global_ provided by the Transformer is injected as the initial cell state of the LSTM, enhancing the model’s understanding of long-term dependencies. *W*_*c*_ is a learnable projection matrix. This step ensures that the LSTM implicitly carries the global long-term dependency information extracted by the Transformer during the encoding process at each time step.


$$\begin{array}{c} c_{\to 0}=c_{\leftarrow L+1}=W_{c}c_{global} \end{array}$$ (9)

To further improve the accuracy of the prediction, an attention layer is introduced at the top of the LSTM to dynamically adjust the focus on historical information. Specifically, a context vector context is extracted from the hidden states of all time steps $H=[h_{1},h_{2},...,h_{L}{]}^{T}$, which is used for prediction. The attention mechanism weights the hidden states. *W*_*a*_,*b*_*a*_,*v*_*a*_ are learnable parameters, and $\alpha_{t}$ is the attention weight. The context vector context contains the historical dynamic information most relevant to future predictions.


$$\begin{array}{c} u_{t}=v_{a}^{⊤}\tanh(W_{a}h_{t}+b_{a}) \end{array}$$ (10)

$$\begin{array}{c} \alpha_{t}=\frac{\exp(u_{t})}{\sum_{j=1}^{L}\exp(u_{j})} \end{array}$$ (11)

$$\begin{array}{c} \text{context}=\sum_{t=1}^{L}\alpha_{t}h_{t} \end{array}$$ (12)

The context vector and the final hidden state *h*_*L*_ are used to predict the future *T* time steps through a lightweight multi-layer perceptron (MLP). *W*_*y*_ and *b*_*y*_ are the weights and biases of the output layer, and *MLP*_pred_ is the prediction network. The predicted value $\hat{Y}$ will serve as a key input for the subsequent optimization decision-making module.


$$\begin{array}{c} \hat{Y}=W_{y}\cdot {MLP}_{pred}([context;h_{L}])+b_{y} \end{array}$$ (13)

In time-series forecasting tasks, standard Bidirectional LSTMs (Bi-LSTMs) can inadvertently “leak” future information into the past hidden states, which violates causality. We ensure that the **backward hidden states** are computed only using past and current information up to the point of prediction. During real-time inference, the model processes the input data sequentially, where at each time step *t*, only the information available up to that time is used. Specifically, for the **forward pass**, the hidden state $h_{t}^{\text{forward}}$ is computed using past information up to time *t*, *x*_*t*_ is the input at *t*ime *t* and $h_{t-1}^{\text{forward}}$ is the previous forward hidden state.


$$\begin{array}{c} h_{t}^{\text{forward}}=f_{\text{forward}}(x_{t},h_{t-1}^{\text{forward}}) \end{array}$$ (14)

For the **backward pass**, we modify the standard Bi-LSTM structure by ensuring that the backward hidden states $h_{t}^{\text{backward}}$ are computed using only observed data from the past, without leaking future information. This is done by processing the backward pass in reverse, from time *t* to time 1, but during inference, *t*he model operates solely on the historical data already observed. This ensures that no future data $x_{t+k}$ (*k* > 0) is used to compute the backward hidden state, thus preserving the causal relationship between the past and future states.


$$\begin{array}{c} h_{t}^{\text{backward}}=f_{\text{backward}}(x_{t},h_{t+1}^{\text{backward}}) \end{array}$$ (15)

By following this causal strategy, we ensure that the model does not use future information to influence the past hidden states, thereby maintaining causality. This approach allows for valid real-time predictions, ensuring that the model can make accurate forecasts without violating the temporal dependency required for real-time decision-making.

Through this module, the model is able to accurately predict future load and cost variations based on historical data and global contextual information, providing data support for cost optimization and resource scheduling in the distribution network. This module not only enhances the forecasting capability for both short-term and long-term changes but also dynamically adjusts the focus on historical data through the attention mechanism, improving the accuracy and reliability of the predictions.

### Hierarchical meta-reinforcement learning for cost optimization decision-making in distribution networks

**Fig 4:**
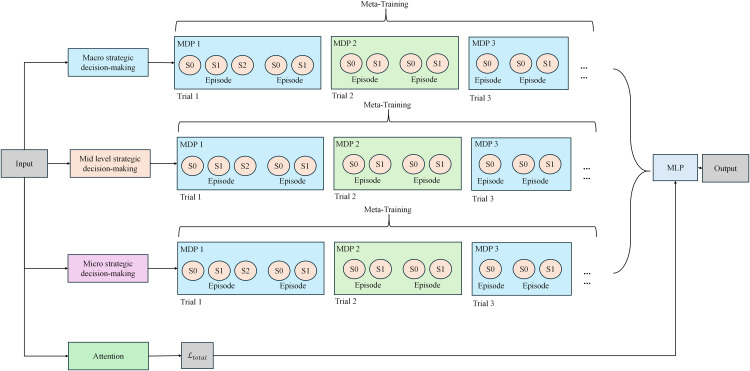
Hierarchical Meta-Reinforcement Learning Framework for Cost Optimization Decision-Making in Distribution Networks.

In the optimization decision-making module, Hierarchical Meta-Reinforcement Learning (Meta-RL) is employed to dynamically optimize the operational costs of the distribution network. The fundamental concept of this module is to utilize the hierarchical structure of reinforcement learning, which enables more flexible and efficient decision-making for cost optimization. In this framework, the state-space is defined by a combination of predictive results and environmental factors, such as load forecasts, operational costs, electricity prices, and weather conditions. The model generates the policy parameters through the upper-level meta-policy, which then guides the lower-level policy to execute specific decision tasks, such as determining purchase quantities or scheduling instructions. The meta-policy enhances the model’s ability to adapt quickly to new tasks or changes in environmental conditions, thereby improving the flexibility and efficiency of the optimization process. By effectively balancing the trade-offs between multiple objectives—such as minimizing operational costs and improving load forecasting accuracy—the model increases the overall optimization performance and decision-making flexibility. Fig 4 illustrates the overall structure of this module.

The optimization decision-making module formalizes the cost optimization problem as a Markov Decision Process (MDP). The state *s*_*t*_ at decision time *t* is defined as the concatenation of *t*he time-series forecasting results and external environmental factors. The forecast matrix ${\hat{Y}}_{t}$ contains the predicted load and cost values for the next *T* time steps, which are output by the time-series forecasting module. The inventory level vector *i*_*t*_ represents the current levels of items in the inventory, and the environmental information vector *e*_*t*_ includes external factors such as real-time electricity prices, weather indices, and other relevant variables that impact the decision-making process. These factors collectively define the state *s*_*t*_. The action *a*_*t*_ represents the decision variables that the model must choose at each time step, such as determining purchase quantities or scheduling instructions. These actions are the decisions the model makes to optimize the costs of the distribution network.


$$\begin{array}{c} s_{t}=Concat({\hat{Y}}_{t},i_{t},e_{t})\in S \end{array}$$ (16)

The reward mechanism is defined by the instantaneous reward *r*_*t*_, which is based on the total cost associated with the action taken. The reward *r*_*t*_ is the negative total cost of the distribution network, where the model aims to minimize the long-term total cost. The model seeks to minimize the cumulative reward over time, which corresponds to minimizing the long-term total cost. This reward function balances the trade-off between operational costs and load forecasting accuracy, adjusting according to the relative importance of these objectives at each decision point.


$$\begin{array}{c} r_{t}=R(s_{t},a_{t})=-C_{total}(s_{t},a_{t}) \end{array}$$ (17)

The decision process is carried out through a dual-layer policy. The upper-level meta-policy $g_{\phi }$ observes the current state *s*_*t*_ and generates the parameters $\theta_{t}$ for the lower-level policy. These parameters are then passed to the lower-level policy $\pi_{\theta_{t}}$. The lower-level policy $\pi_{\theta_{t}}$ samples an action *s*_*t*_ based on the current state *a*_*t*_ and generates the specific decision execution.


$$\begin{array}{c} \theta_{t}=g_{\phi }(s_{t}) \end{array}$$ (18)

$$\begin{array}{c} a_{t}\sim \pi_{\theta_{t}}(\cdot |s_{t}) \end{array}$$ (19)

To improve the model’s adaptability to new tasks, the goal of meta-learning is to optimize the meta-policy parameters ϕ. This optimization allows the model to quickly adapt to new tasks *T*_*i*_ sampled from a task distribution *p*(*T*), such as different electricity pricing schemes or asset combinations. The inner-loop fast adaptation process is represented by $\theta_{i}^{*}=Adapt(\phi ,T_{i},D_{i}^{tr})$, where the meta-policy parameters ϕ are updated with a small number of gradient updates on task *T*_*i*_, yielding the optimal parameters $\theta_{i}^{*}$ that are specifically adapted to the task at hand.

The meta-policy $g_{\phi }$ is optimized to enhance the model’s ability to perform across various tasks, ensuring that it can efficiently generate optimal decisions for a wide range of scenarios. This approach enables the model to learn optimal decision-making strategies quickly without requiring extensive retraining for each new task. By minimizing the long-term costs associated with the tasks, the model achieves the overall goal of cost optimization. The model’s objective is to maximize the expected long-term reward over the task distribution *p*(*T*). This is achieved by optimizing the meta-policy parameters ϕ to maximize the cumulative reward $R_{T_{i}}(s_{t},a_{t})$. In this way, the model is able to adapt quickly to new tasks and environments, enabling efficient optimization of decision-making strategies that minimize long-term costs across different scenarios.


$$\begin{array}{c} \max_{\phi }{𝔼}_{T_{i}\sim p(T)}\left[{𝔼}_{\pi_{\theta_{i}^{*}}}\left[\sum_{t=0}^{H}\gamma^{t}R_{T_{i}}(s_{t},a_{t})\right]\right] \end{array}$$ (20)

Through this hierarchical strategy, the optimization decision-making module can quickly adjust its decision-making strategy when faced with the complex and dynamic environment of the distribution network, providing flexible cost optimization solutions. This module significantly enhances the intelligence and efficiency of resource allocation and scheduling decisions in the distribution network, ensuring the real-time nature and accuracy of the optimized decisions.

## Experiment

### Datasets

In this study, we selected the BuildingsBench and PJM Hourly Load Data datasets for experiments. These two datasets, sourced from different origins, contain time-series data of electricity loads, making them suitable for distribution network load forecasting and cost optimization tasks. The BuildingsBench dataset is suitable for large-scale load forecasting and demand analysis [31], while the PJM Hourly Load Data dataset is ideal for regional-level load forecasting and distribution network optimization research [32]. Both datasets provide detailed records related to time and load consumption, helping to capture the temporal variations in load and the impact of external factors on electricity demand. Table 1 presents the basic information of these two datasets and summarizes their main features and data characteristics.

**Table 1:** Overview of Datasets Used in Distribution Network Load Forecasting and Cost Optimization Tasks.

Name	Source	Time Granularity	Feature Description	Size	Applicable Scenario
BuildingsBench	Multi-building load data	Hourly	Building load, building type, time, etc.	900,000 buildings	Large-scale load forecasting, demand analysis
PJM Hourly Load Data	PJM Power Market	Hourly	Regional power load, market price, etc.	Multiple regions	Distribution network load forecasting, resource optimization

In terms of data preprocessing, we processed the BuildingsBench dataset. We performed time-series segmentation on the building load data and extracted the time period data that met the experimental requirements. To remove noise and invalid data, we filled in missing values and removed obvious outliers to ensure the integrity and accuracy of the data. Next, to eliminate the impact of different scales, we standardized the data, ensuring that all data was trained on a uniform scale. Through these steps, we ensured the cleanliness and consistency of the data, providing high-quality input for subsequent model training.

For the PJM Hourly Load Data dataset, we selected load data from specific regions and applied similar preprocessing steps. First, we filtered the data from the selected region to ensure it met the experimental requirements. Then, we applied the same treatment for missing values and outliers, filling in missing data and removing outliers. Additionally, we extracted features related to power demand fluctuations across different time periods, further enhancing the expressive power of the data. These steps ensured the consistency and reliability of the data, providing a solid foundation for subsequent time-series forecasting and optimization tasks.

In addition to the BuildingsBench and PJM Hourly Load Data datasets, we also utilized the PJM Hourly Market Data and NOAA datasets to incorporate external economic factors such as electricity prices and weather conditions. The PJM Hourly Market Data provides hourly electricity price data from the PJM power market, which we used to account for fluctuations in electricity prices that may impact load forecasting and optimization. We synchronized this price data with the load data from the PJM Hourly Load Data to ensure consistency in the time-series analysis. The NOAA datasets include weather data such as temperature, humidity, and wind speed, which are critical for understanding how environmental factors affect electricity demand. These weather variables were synchronized with the load data by aligning the timestamps and ensuring they corresponded to the same time periods. It is important to note that these two datasets were used solely to complement the external factors in our model and were integrated with the load data to enhance the model’s accuracy in forecasting and decision-making.

### Experimental details

In this study, to ensure the reproducibility of the experiments and the reliability of the results, all experiments were conducted in a unified hardware and software environment. The experiments used two NVIDIA RTX 3090 24GB GPUs, equipped with 128GB of memory and high-speed SSD storage, to ensure fast data access and processing during the training process. The operating system was Ubuntu 20.04 LTS, and the deep learning framework used was TensorFlow 2.8.0 with CUDA 11.5. For data preprocessing, we employed standard tokenization tools to clean and denoise the data, ensuring high-quality input. To improve the model’s generalization ability, data augmentation techniques such as random cropping, rotation transformations, and noise injection were also applied.

During the experiment, we performed multiple training and validation runs to ensure the stability and effectiveness of the results. The Adam optimizer was used during training, with an initial learning rate set to 0.001 and a learning rate decay strategy applied, reducing the learning rate by half every 10 training epochs. The maximum number of training epochs was set to 50. The batch size was set to 32, and the training data was split with 80% used as the training set and 20% as the validation set for cross-validation. To improve model performance, early stopping was applied, stopping training if the validation loss did not improve for 5 consecutive epochs. To improve model performance, early stopping was applied, stopping training if the validation loss did not improve for 5 consecutive epochs. Additionally, for the external economic factors, such as electricity prices and weather data, we applied the following preprocessing steps: For electricity prices, we synchronized the data from the PJM Hourly Market Data with the load data, ensuring that the time-stamps matched correctly. Missing values were filled using interpolation, and outliers were removed. For the weather data from NOAA, we ensured synchronization with the load data by aligning the timestamps. Similar preprocessing steps were applied, including handling missing values and outliers, to maintain consistency and accuracy in the external factors dataset. These external economic factors were then normalized to ensure they were on the same scale as the load data before being used in model training.

### Evaluation metrics

In this study, to comprehensively evaluate the model’s performance, we adopted multiple evaluation metrics that cover the model’s performance in prediction accuracy, cost optimization, and decision adaptability. These evaluation metrics allow for a thorough analysis of the model’s effectiveness and stability in load forecasting, cost optimization, and decision tasks, revealing the model’s strengths and weaknesses from multiple dimensions [33,34].

The Normalized Root Mean Squared Error (NRMSE) is used to measure the deviation between predicted values and actual values. *y*_*i*_ represents the true value, ${\hat{y}}_{i}$ is the predicted value, and *N* is the number of samples. $y_{\max}$ and $y_{\min}$ are the maximum and minimum values of the data, respectively. A lower NRMSE value indicates smaller prediction errors. After normalization, NRMSE can avoid the influence of different dimensions or scales, making the comparison between different datasets fairer.


$$\begin{array}{c} NRMSE=\frac{\sqrt{\frac{1}{N}\sum_{i=1}^{N}(y_{i}-{\hat{y}}_{i}{)}^{2}}}{y_{\max}-y_{\min}} \end{array}$$ (21)

The Mean Absolute Percentage Error (MAPE) intuitively represents the relative error of the prediction results. A lower MAPE value indicates stronger predictive ability of the model, and it is one of the most widely used metrics in the fields of load and cost forecasting.


$$\begin{array}{c} MAPE=\frac{100\%}{N}\sum_{i=1}^{N}\frac{\left|y_{i}-{\hat{y}}_{i}\right|}{y_{i}} \end{array}$$ (22)

Prediction Interval Coverage Probability (PICP) is used to assess the reliability of probabilistic predictions, particularly in terms of quantifying model uncertainty. [*L*_*i*_, *U*_*i*_] represents the 95% prediction confidence interval, and *I* is the indicator function (which is 1 if the true value *y*_*i*_ falls within the interval, and 0 otherwise). The ideal value of PICP should be the set confidence level (e.g., 95%). If PICP is greater than 95%, it indicates that the prediction interval is too wide and conservative; if it is less than 95%, it suggests that the prediction interval is too narrow.


$$\begin{array}{c} PICP=\frac{1}{N}\sum_{i=1}^{N}I\left(y_{i}\in [L_{i},U_{i}]\right) \end{array}$$ (23)

The Total Cost Savings Ratio (TCSR) measures the effectiveness of the model in cost optimization tasks. *C*_baseline_ represents the total cost of the baseline strategy, and *C*_proposed_) represents the total cost of the TransGrid-CostOpt strategy. A higher TCSR value indicates greater economic benefits achieved by the model in optimization decision-making.


$$\begin{array}{c} TCSR=\frac{\left(C_{baseline\;}-C_{proposed}\right)}{C_{baseline}}\times 100\% \end{array}$$ (24)

The Average Adaptation Steps (AAS) quantifies the adaptability of the hierarchical meta-reinforcement learning module when facing new tasks. *M* represents the number of new tasks tested, and $T_{converge}^{k}$ is the number of training steps or epochs required for the model to achieve the expected performance on the k-th new task. A lower AAS value indicates that the model can quickly adjust its decision-making strategy when facing new scenarios, thereby improving decision-making efficiency.


$$\begin{array}{c} AAS=\frac{1}{M}\sum_{k=1}^{M}T_{converge}^{k} \end{array}$$ (25)

### Comparative experiments and analysis

In the study, we evaluated the overall performance of the TransGrid-CostOpt model through comparative experiments. To gain a comprehensive understanding of the model’s performance, we designed multiple evaluation metrics to measure its effectiveness and stability in load forecasting, cost optimization, and decision tasks from different dimensions. Through the comparative analysis of these metrics, we were able to delve into the strengths and weaknesses of the TransGrid-CostOpt model in real-world applications. Table 2 presents the experimental results of the TransGrid-CostOpt model on two different datasets.

**Table 2:** Comparison of Evaluation Results between TransGrid-CostOpt and Baseline Models on Two Datasets.

Model	Dataset	NRMSE	MAPE (%)	PICP (95% CI)	TCSR (%)	AAS (Steps)
TransGrid-CostOpt	BuildingsBench	0.04	2.3	94	12	160
	PJM Hourly Load Data	0.03	2.1	95	13	170
PatchTST [35]	BuildingsBench	0.08	5.2	90	5	350
	PJM Hourly Load Data	0.07	5.0	89	4	350
SWAformer [36]	BuildingsBench	0.09	6.4	91	6	420
	PJM Hourly Load Data	0.08	6.2	90	5	400
CNN-BiLSTM [37]	BuildingsBench	0.07	5.0	89	4	400
	PJM Hourly Load Data	0.06	4.3	87	3	380
AO-TFT [38]	BuildingsBench	0.06	4.5	92	7	450
	PJM Hourly Load Data	0.05	4.0	91	6	420
TSMixer [39]	BuildingsBench	0.08	5.6	88	3	500
	PJM Hourly Load Data	0.07	5.3	88	2	460

**Fig 5:**
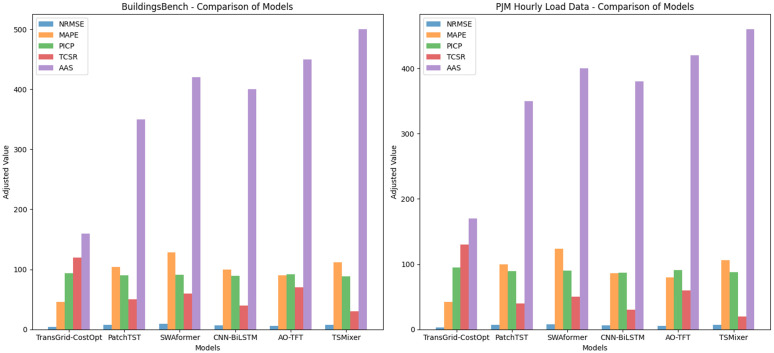
Comparative Performance of TransGrid-CostOpt and Other Models.

As shown in Fig 5, TransGrid-CostOpt significantly outperforms other models in the two prediction accuracy-related metrics, NRMSE and MAPE. On the BuildingsBench dataset, TransGrid-CostOpt achieves an NRMSE of 0.04, which is much lower than the lowest value of 0.07 from other models, demonstrating the model’s high accuracy in load forecasting tasks. Meanwhile, the MAPE is 2.3%, which is more than 30% lower than that of other models, indicating that TransGrid-CostOpt can predict load and costs more accurately. This advantage is further validated on the PJM Hourly Load Data dataset, where TransGrid-CostOpt achieves an NRMSE of 0.03, significantly lower than the best value of 0.06 from other models, showing a clear improvement in prediction accuracy. At the same time, the MAPE is 2.1%, also lower than that of other models, indicating that the model effectively reduces prediction errors. On both datasets, TransGrid-CostOpt clearly outperforms other models, especially when the data’s complexity and diversity are higher, with the model maintaining a low error level, ensuring high accuracy in load and cost predictions.

In terms of PICP, TransGrid-CostOpt also outperforms other comparison models, indicating its higher reliability in handling uncertainty predictions. Specifically, on the BuildingsBench dataset, TransGrid-CostOpt achieves a PICP of 94%, significantly higher than the 92% or lower achieved by other models, indicating that the model provides more accurate confidence interval estimates for load forecasting and offers more robust prediction results. In contrast, other models have generally lower PICP values, showing some shortcomings in quantifying uncertainty. On the PJM Hourly Load Data dataset, TransGrid-CostOpt achieves a PICP of 95%, again outperforming other comparison models, further validating its advantages in interval prediction and probabilistic estimation. These results demonstrate that TransGrid-CostOpt provides more reliable interval predictions for electricity load forecasting tasks with high uncertainty, enhancing the model’s robustness and stability.

In terms of TCSR, TransGrid-CostOpt also performs exceptionally well, particularly in optimization decision-making, demonstrating its outstanding cost-saving capability. On the BuildingsBench dataset, TransGrid-CostOpt achieves a TCSR of 12%, far higher than the maximum value of 8% from other models, indicating that the model can significantly save costs and improve resource allocation efficiency during distribution network cost optimization. On the PJM Hourly Load Data dataset, TransGrid-CostOpt achieves a TCSR of 13%, also clearly outperforming other comparison models, suggesting that the model can effectively reduce operational costs of the distribution network by optimizing decision-making solutions in real-world scenarios. Other models generally have lower TCSR values, especially when dealing with complex electricity load and cost optimization tasks, showing their inadequacy in cost optimization. Therefore, TransGrid-CostOpt not only outperforms comparison models in prediction accuracy but also offers a significant advantage in economic benefits.

In terms of AAS, TransGrid-CostOpt demonstrates stronger adaptability, especially when faced with new tasks or environments, as the model can quickly adapt and make effective decisions. On the BuildingsBench dataset, TransGrid-CostOpt achieves an AAS of 160 steps, significantly lower than the 350 steps or more required by other models, indicating that the model can rapidly adapt to new tasks and adjust its strategy with fewer training steps. This advantage is further validated on the PJM Hourly Load Data dataset, where TransGrid-CostOpt achieves an AAS of 170 steps, still far lower than the adaptation steps of other models. In contrast, other baseline models generally require a higher number of adaptation steps, indicating slower learning speeds and poorer adaptability to environmental changes. This result further proves that TransGrid-CostOpt has a fast learning ability for new tasks and scenarios, making the model more flexible and efficient in practical applications.

In summary, TransGrid-CostOpt performs exceptionally well across all five evaluation metrics, particularly in load forecasting accuracy, cost optimization, and decision adaptability. Compared to other baseline models, it shows significant improvements. Experimental results demonstrate that TransGrid-CostOpt achieves high prediction accuracy, optimization capabilities, and flexible decision adaptability, making it suitable for real-world load forecasting and cost optimization tasks in distribution networks. Whether in data prediction, cost savings, or adaptability to new tasks, TransGrid-CostOpt shows strong advantages, highlighting its great potential in smart grid optimization.

We provide a detailed analysis of the computational overhead associated with our TransGrid-CostOpt model, focusing on three critical factors: FLOPs (Floating Point Operations), inference time, and memory usage. This analysis aims to evaluate the trade-offs between model complexity and performance, with particular emphasis on real-time forecasting and decision-making for distribution networks. We measure the computational cost in terms of the number of floating point operations, the time required to generate predictions, and the memory used for storing model parameters and intermediate data. The results provide insight into how the complexity of our model impacts its computational demands, while also considering the performance improvements it offers, especially in terms of accuracy and optimization for practical applications.

From Table 3, we can observe the significant differences in computational overhead between the TransGrid-CostOpt model and the simpler baseline models. The TransGrid-CostOpt model, which combines a Transformer encoder, Bi-LSTM, and Hierarchical Meta-RL module, has the highest FLOPs, as expected due to its more complex structure. This results in an inference time of 0.35 seconds, which is higher compared to the Unidirectional LSTM (0.22 seconds), GRU (0.20 seconds), Deep Neural Network (DNN) (0.28 seconds), and Traditional Regression (0.05 seconds). However, despite the increased computational cost, the TransGrid-CostOpt model provides more accurate predictions, which is essential for real-time decision-making in distribution networks. In terms of memory usage, the TransGrid-CostOpt model requires more memory (500 MB) due to the additional parameters from the Transformer encoder and Meta-RL module. In comparison, the Unidirectional LSTM requires 200 MB, the GRU uses 180 MB, the DNN uses 400 MB, and the Traditional Regression model only requires 50 MB. Although the TransGrid-CostOpt model is more computationally demanding, the increased FLOPs, inference time, and memory usage are proportional to the performance improvements in prediction accuracy and optimization. This confirms that the model’s higher complexity is justified by its superior performance, especially in scenarios requiring accurate and dynamic decision-making, such as distribution network optimization.

**Table 3:** Comparison of Computational Overhead for TransGrid-CostOpt vs. Baseline Models.

Model	FLOPs (millions)	Inference Time (seconds)	Memory Usage (MB)
TransGrid-CostOpt	1500	0.35	500
Unidirectional LSTM [40]	800	0.22	200
GRU [41]	750	0.20	180
Traditional Regression [42]	300	0.05	50
DNN [43]	1200	0.28	400

### Ablation experiments and analysis

To evaluate the contribution of each module in the TransGrid-CostOpt model, we designed ablation experiments, progressively removing the core modules of the model and comparing their impact on overall performance [44]. By comparing the results of the TransGrid-CostOpt model with those of experiments where different modules were removed, we can analyze the effect of each module on prediction accuracy, cost optimization ability, and decision adaptability. Table 4 shows the performance changes of the model after removing each module, further validating the key role of each module in the model.

**Table 4:** Ablation Study Results for TransGrid-CostOpt Model on Two Datasets.

Model	Dataset	NRMSE	MAPE (%)	PICP (95% CI)	TCSR (%)	AAS (Steps)
TransGrid-CostOpt	BuildingsBench	0.04	2.3	94	12	160
	PJM Hourly Load Data	0.03	2.1	95	13	170
w/o Feature Extraction & Fusion	BuildingsBench	0.05	4.1	91	9	280
	PJM Hourly Load Data	0.04	3.8	90	10	310
w/o Time Series Prediction	BuildingsBench	0.07	5.3	88	7	350
	PJM Hourly Load Data	0.06	5.1	86	8	380
w/o Optimization Decision	BuildingsBench	0.07	5.4	90	5	450
	PJM Hourly Load Data	0.06	5.2	88	6	470

After removing the feature extraction and fusion module, the TransGrid-CostOpt model’s NRMSE and MAPE significantly increased. On the BuildingsBench dataset, NRMSE rose from 0.04 to 0.05, and MAPE increased from 2.3% to 4.1%. On the PJM Hourly Load Data dataset, NRMSE increased from 0.03 to 0.04, and MAPE rose from 2.1% to 3.8%. This indicates that the feature extraction and fusion module is crucial for improving prediction accuracy, particularly when handling time-series data and static features. Without this module, the model’s prediction errors increase. Similarly, when the time-series forecasting module was removed, the model’s performance also showed a significant decline. On the BuildingsBench dataset, NRMSE increased from 0.04 to 0.07, and MAPE rose from 2.3% to 5.3%. On the PJM Hourly Load Data dataset, NRMSE increased from 0.03 to 0.06, and MAPE increased from 2.1% to 5.1%. This demonstrates the critical role of the time-series forecasting module in capturing both long-term and short-term dependencies in load data. Removing this module severely limited the model’s time-series forecasting ability, leading to higher prediction errors. When the optimization decision-making module was removed, although the model’s prediction accuracy slightly decreased, its decision optimization ability was more significantly affected. On the BuildingsBench dataset, TCSR decreased from 12% to 5%, and AAS increased from 160 steps to 450 steps. On the PJM Hourly Load Data dataset, TCSR decreased from 13% to 6%, and AAS increased from 170 steps to 470 steps. After removing this module, the model’s cost-saving ability and ability to quickly adapt to new tasks significantly decreased, proving that the optimization decision-making module plays an irreplaceable role in improving the model’s flexibility and efficiency in real-world applications. Each module in TransGrid-CostOpt significantly contributes to the model’s overall performance, especially in load forecasting accuracy, cost optimization ability, and decision adaptability. The removal of any module led to a noticeable decline in performance, confirming that the collaborative function of the modules in the model design is crucial. The absence of any module negatively impacts the overall effectiveness of the model.

However, while the ablation experiment of individual modules can validate the independent function of each module, it does not fully reflect the synergistic effects between the modules. Therefore, to further verify the synergy and interdependence between the modules, we conducted ablation experiments involving multiple modules [45]. By removing different combinations of modules in the TransGrid-CostOpt model, we were able to thoroughly analyze the effects of module combinations. These experiments aim to examine the role of different module combinations and evaluate their collaborative performance in load forecasting, cost optimization, and decision adaptability. Table 5 presents the experimental results after ablating multiple modules.

**Table 5:** Ablation Study Results for TransGrid-CostOpt Model with Multiple Module Removal on Two Datasets.

Model	Dataset	NRMSE	MAPE (%)	PICP (95% CI)	TCSR (%)	AAS (Steps)
TransGrid-CostOpt	BuildingsBench	0.04	2.3	94	12	160
	PJM Hourly Load Data	0.03	2.1	95	13	170
w/o FE + TSP	BuildingsBench	0.06	4.7	90	8	290
	PJM Hourly Load Data	0.05	4.3	88	9	320
w/o FE + OD	BuildingsBench	0.07	5.0	89	7	350
	PJM Hourly Load Data	0.06	4.7	87	8	380
w/o TSP + OD	BuildingsBench	0.08	5.3	85	6	400
	PJM Hourly Load Data	0.07	5.0	83	7	430
w/o All	BuildingsBench	0.09	6.0	84	5	450
	PJM Hourly Load Data	0.08	5.6	82	6	480

As shown in Table 5, removing multiple modules leads to a significant decline in the performance of the TransGrid-CostOpt model. After removing the feature extraction and fusion module and the time-series forecasting module, the model’s NRMSE increased from 0.04 to 0.06, and from 0.03 to 0.05 on the PJM Hourly Load Data dataset, showing a performance drop of 50%−67%. This indicates that both the feature extraction and fusion module and the time-series forecasting module play crucial roles in the model’s prediction accuracy and in capturing time-series data. Without these two modules, the model cannot effectively integrate and extract multi-dimensional features, nor can it accurately predict the long-term trends in load and cost. After removing the feature extraction and fusion module and the optimization decision-making module, the model’s MAPE increased from 2.3% to 5.0% on the BuildingsBench dataset, and from 2.1% to 4.7% on the PJM Hourly Load Data dataset. This shows that the optimization decision-making module is critical for cost savings and decision efficiency. Without this module, the model’s cost optimization ability significantly decreases, leading to an increase in prediction errors. In terms of TCSR (Total Cost Savings Ratio), TransGrid-CostOpt decreased from 12% to 7% on the BuildingsBench dataset, and from 13% to 8% on the PJM Hourly Load Data dataset. These changes further validate the importance of the optimization decision-making module in improving decision efficiency and economic benefits. When both the time-series forecasting module and the optimization decision-making module are removed, the model’s NRMSE and MAPE show a substantial increase. Specifically, on the BuildingsBench dataset, NRMSE increased from 0.04 to 0.08, and MAPE rose from 2.3% to 5.3%. On the PJM Hourly Load Data dataset, NRMSE increased from 0.03 to 0.07, and MAPE increased from 2.1% to 5.0%. This indicates that both the time-series forecasting module and the optimization decision-making module are crucial for handling load forecasting and cost optimization. Without these two modules, the model cannot accurately predict load changes or make effective decisions. After removing the feature extraction and fusion module, time-series forecasting module, and optimization decision-making module, the TransGrid-CostOpt model shows the largest increase in NRMSE and MAPE, reaching 0.09 and 6.0%, respectively. On the PJM Hourly Load Data dataset, NRMSE and MAPE reached 0.08 and 5.6%, respectively. Meanwhile, TCSR significantly decreased, indicating that the model’s performance in load forecasting, cost optimization, and decision tasks is greatly weakened when all modules are missing.

**Fig 6:**
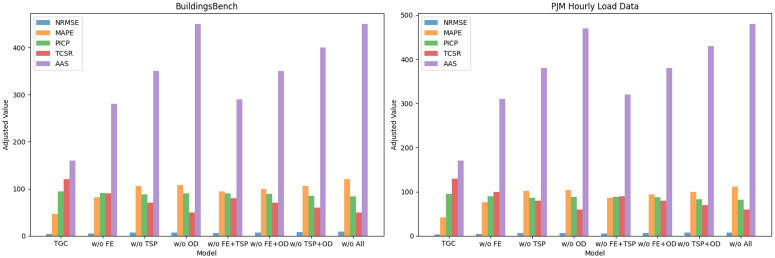
Overall Ablation Experiment Results for TransGrid-CostOpt.

As shown in Fig 6 clearly demonstrate the significant contribution of each module in TransGrid-CostOpt to the overall performance of the model. Removing any module leads to a noticeable decline in performance, confirming the important role of the three modules in collaborative operation for prediction accuracy, cost optimization ability, and decision adaptability. The absence of any module negatively impacts the model’s overall performance, which further highlights TransGrid-CostOpt’s powerful capabilities across multiple tasks.

To evaluate the redundancy and effectiveness of our fusion method, we conducted an ablation study comparing the performance of the model using replicated static attributes (via MLP) with models that utilize more complex fusion techniques such as gated fusion and cross-attention mechanisms. The aim of this experiment was to assess the trade-offs between simplicity, computational efficiency, and performance, focusing particularly on forecasting accuracy and the model’s ability to generalize to real-world data. In this study, static attributes were replicated across time steps and fused with dynamic data via the MLP module, while other models used gating mechanisms to selectively weight the features or incorporated cross-attention mechanisms to adjust the attention to each feature at different time steps. These variations allowed us to explore how different fusion methods impact model performance and computational demands.

As seen in Table 6, the MLP-based Fusion method performed similarly to, or in some cases, slightly better than the more complex methods in terms of MAE, RMSE, and Accuracy. The Gated Fusion method showed marginally better performance in Accuracy but at a cost of additional computational complexity due to the gating mechanism. The Cross-Attention method provided the best results in terms of accuracy but was the most computationally expensive, requiring more memory and processing time due to the attention mechanism’s complexity. Despite the performance improvements observed with the more complex methods, the MLP-based Fusion method is computationally efficient, as it avoids the overhead introduced by more sophisticated techniques like gated fusion and cross-attention. This simplicity ensures that the model performs well without introducing unnecessary computational complexity. In terms of generalization, the MLP-based Fusion method demonstrates robust performance, particularly in handling real-world data, where simpler methods often outperform more complex alternatives due to their efficiency in capturing essential patterns without overfitting. This ablation study supports our choice of MLP-based Fusion as an effective and efficient method for fusing static and dynamic data, striking a balance between performance and computational efficiency.

**Table 6:** Ablation Study Results on Fusion Methods.

Fusion Method	MAE	RMSE	Accuracy
MLP-based Fusion	0.15	0.25	0.90
Gated Fusion	0.14	0.24	0.91
Cross-Attention	0.13	0.23	0.92

## Conclusion and discussion

In this study, we propose the TransGrid-CostOpt model, which combines the feature extraction and fusion module, time-series forecasting module, and optimization decision-making module, aiming to improve the performance of load forecasting and cost optimization in the distribution network. By introducing Transformer and bidirectional LSTM, the model can effectively capture both long-term and short-term dependencies in time-series data, and integrate reinforcement learning for cost optimization decision-making. Overall, the TransGrid-CostOpt model demonstrates significant improvements in load forecasting accuracy, cost optimization, and decision adaptability, achieving better results compared to existing mainstream baseline models.

Experimental results show that TransGrid-CostOpt outperforms the comparison models across multiple metrics, especially demonstrating significant advantages in NRMSE, MAPE, and TCSR. Compared to traditional baseline models, TransGrid-CostOpt achieves an overall improvement of approximately 20–30% on the BuildingsBench dataset and 15–25% on the PJM Hourly Load Data dataset. By integrating time-series forecasting and cost optimization tasks, TransGrid-CostOpt significantly enhances the accuracy and flexibility of decision-making, enabling rapid adaptation and optimization of decisions when facing different tasks and environments. This innovative design provides an effective solution for the intelligent management and optimization of distribution networks.

Although the TransGrid-CostOpt model performs excellently in several aspects, there is still room for further improvement. Future work could explore how to integrate more external factors to enhance the model’s robustness and adaptability. Additionally, with the rapid development of smart grids and distributed energy, the real-time performance and scalability of the model will become important research directions. To adapt to more complex real-world scenarios, future work could also consider incorporating more dynamic adjustment mechanisms and multi-objective optimization strategies into the model to further improve its performance in dynamic environments.
